# Supplementary material for: Identification of photoperiod-induced specific miRNAs in the adrenal glands of Sunite sheep (Ovis aries)
Source: Front Vet Sci. 2022 Jul 22;9:888207. doi: 10.3389/fvets.2022.888207 (PMC9354845; doi:10.3389/fvets.2022.888207)
Supplement: Supplementary file 1 [file Data_Sheet_1.ZIP › Supplementary file/miRDeep2 file.zip!/miRDeep2 file/Novel_Structure/LP42A3/Novel_214.pdf]

Provisional ID	:	NC_019478.2_21460
Score total	:	1.9
Score for star read(s)	:	-1.3
Score for read counts	:	0
Score for mfe	:	1.6
Score for randfold	:	1.6
Score for cons. seed	:	
Total read count	:	41
Mature read count	:	41
Loop read count	:	0
Star read count	:	0

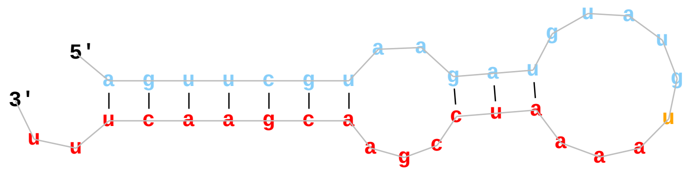

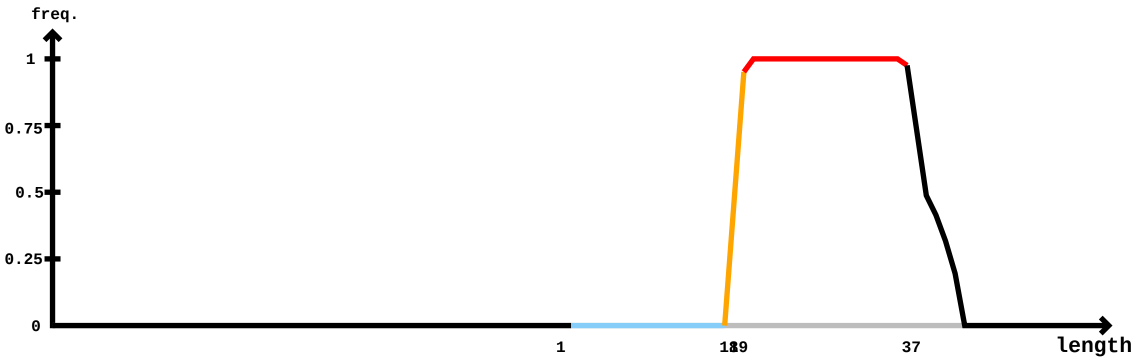

Star Mature

```
5'- gcagaggaaggaaggagggggugucugagaggguauugggcuugggccagaaaguucguaagauguauguaaaauccgaacgaacuuuuugggcccacccaguauuuc   -3'    exp
    .((((.(..............).))))...((((((((((..((((((((((((((((..(((.........)))...))))))))))).)))))))))))))))).    reads    mm    sample
    ....................................................................uaaaauccgaacgaacuuuuug.................    1    0    seq
    ....................................................................uaaaauccgaacgaacuuuuugg................    1    0    seq
    .....................................................................aaaauccgaacgaacuu.....................    1    0    seq
    .....................................................................aaaauccgaacgaacuuu....................    16   0    seq
    .....................................................................aaaauccgaaUgaacuuuu...................    1    1    seq
    .....................................................................aaaaucUgaacgaacuuuu...................    3    1    seq
    .....................................................................aaaauccgaacAaacuuuuu..................    3    1    seq
    .....................................................................aaaauccAaacgaacuuuuug.................    1    1    seq
    .....................................................................aaaauccgaaUgaacuuuuug.................    1    1    seq
    .....................................................................aaaauccgaaUgaacuuuuugg................    4    1    seq
    .....................................................................aaaauccgaacgaacuuuuuggg...............    7    0    seq
    ......................................................................aaauccgaacgaacuuuuug.................    1    0    seq
    ......................................................................aaauccgaacgaacuuuuuggg...............    1    0    seq
```
